# TALEN-mediated genome-editing approaches in the liverwort *Marchantia polymorpha* yield high efficiencies for targeted mutagenesis

**DOI:** 10.1186/s13007-017-0167-5

**Published:** 2017-03-29

**Authors:** Sarah Kopischke, Esther Schüßler, Felix Althoff, Sabine Zachgo

**Affiliations:** Botany Department, School of Biology/Chemistry, Osnabrueck University, Osnabrueck, Germany

**Keywords:** TALEN, Genome editing, *Marchantia polymorpha*, Mp*NOP1*

## Abstract

**Background:**

The liverwort *Marchantia polymorpha* occupies a crucial position in land plant evolution and provides the opportunity to investigate adaptations to a terrestrial plant life style. Marchantia reverse genetic analyses have thus far been conducted by employing a homologous recombination approach, which yields an efficiency of around 3%. Availability of the characterized and suitable endogenous Mp*EF1α* promoter prompted us to establish the TALEN gene targeting technique for Marchantia.

**Results:**

Here, two different TALEN techniques, using custom and self-assembled TALEN constructs, were applied and compared. The Mp*NOP1* gene was selected as a candidate gene, as the respective knockout mutant has been shown to lack air chamber formation, representing an easily traceable phenotype. We demonstrate that both TALEN approaches are successful in Marchantia yielding high gene targeting efficiencies of over 20%. Investigation of selected G1 up to G4 generations proved the stability of the knockout mutants. In 392 analyzed T1 plants, no additional phenotypes were observed and only one chimeric knockout plant was detected after an extended cultivation period. Interestingly, two out of the 24 sequenced mutants harbored indels causing in-frame mutations and revealed novel Mp*nop1*-related phenotypes. This demonstrates the potential to detect crucial amino acids and motives of targeted proteins, which is of special interest for essential genes where full knockouts are lethal. The FastTALE™ TALEN assembly kit enables the rapid assembly and ligation of the TALEN arms within half a day. For transformations, custom and assembled constructs were subcloned into Marchantia binary vectors possessing the Mp*EF1α* promoter.

**Conclusion:**

Considering time, costs and practicability, the assembly TALEN approach represents a rapid and highly efficient gene targeting system to generate Marchantia knockout mutants, which can be further adapted for future advanced genome-editing applications.

## Background

*Marchantia polymorpha* is a complex thalloid liverwort with a haploid gametophyte-dominant life cycle, where the sporophyte is largely dependent upon the gametophyte [1]. Liverworts, together with mosses and hornworts, belong to the group of bryophytes which diverged approximately 470 Mya from the earliest terrestrial plants [2]. Representing one of the earliest land plant lineages, liverworts are therefore a key group to investigate the genetic basis of evolutionary effects associated with the transition from water to land and the origin of land plant innovations. *Marchantia polymorpha* has a long research history with histological studies dating back into the last centuries [3]. Pioneering work has been conducted on the gene content and organization of plant mitochondria, chloroplasts and the Y sex chromosome [4–7]. Whereas ancient polyploidization events increased the genome content in the moss model organism *Physcomitrella patens* [8], evidence for increased chromosome numbers is lacking for liverworts such as Marchantia that have likely experienced a low rate of chromosomal evolution. These findings, together with the relatively small genome size of about 280 Mb [9] and an easy cultivation in the laboratory spurred the development of molecular techniques to establish Marchantia as a basal land plant model organism. Robust and rapid Agrobacterium mediated transformation techniques enable small and large screens of transgenic T1 populations [10–13]. Analysis of nuclear and organellar *M. polymorpha* genomes is conducted under the Community Sequencing Program at the Joint Genome Institute (DOE-JGI: http://jgi.doe.gov/whysequence-a-liverwort/), further enhancing the attractiveness of this bryophyte model system.

For reverse genetic approaches, two gene-targeting techniques have been established to generate knockout mutants. Targeted genome modification by homologous recombination (HR) is highly effective in *P. patens*, but more difficult in other plant species [14]. To enhance the knockout-rates in Marchantia, a positive/negative selection system has been established, which employs the hygromycin resistance gene along with the diphtheria toxin A fragment [15]. Mp*NOP1* was chosen as a candidate gene as the knockout mutant lacks air chamber formation and can therefore be easily identified by phenotypic screening [16]. The HR approach yielded an efficiency of about 3% [15] and has been successfully applied for the generation of several non-essential Marchantia knockout mutants, comprising Mp*GI*, Mp*FKF* [17], Mp*PHOT* [18] and Mp*TAA* [19].

In the past few years, targeted genome editing by employing artificial nucleases paved the ground for rapidly modifying genomes with high efficiencies in a large variety of organisms [20–23]. Most recently, mainly the TALEN (transcription activator-like effector nucleases) and CRISPR/Cas9 (clustered regularly interspaced short palindromic repeat/CRISPR-associated) genome-editing techniques have been successfully applied to edit plant genomes [24–26]. Both systems use endonucleases to create double-strand breaks (DSBs) at a selected target site. Subsequent screenings aim to identify transgenic plants in which imprecise non-homologous end-joining (NHEJ) events introduced deletions, insertions or larger chromosomal replacements at the target site enabling the selection of mutants, which can no longer synthesize functional proteins.

The CRISPR/Cas9 system, originally providing an adaptive immune apparatus for prokaryotes, has been applied for reverse genetic studies in different monocot and eudicot species [24, 27, 28]. In 2014, Sugano and coworkers adopted the CRISPR/Cas9 system for Marchantia [29]. CRISPR/Cas9 sequence specificity is conferred by a short 20 nt sequence that mediates via Watson–Crick base pairing binding to the target site. This sequence is part of a chimeric single guide RNA (sgRNA) that directs the Cas9 endonuclease protein to cleave the targeted DNA thereby generating blunt ends [21].

TALENs are a combination of the FokI endonuclease and the TAL effector (TALE) DNA-binding domain derived from the transcription activator-like effectors from *Xanthomonas spec.* [20, 22]. Binding specificity is conveyed by multiple, nearly identical repeats comprising 33–35 aa sequences which differ only at the positions 12 and 13, called repeat-variable di-residue (RVDs). Each repeat binds to a certain single nucleotide (HD = C, NG = T, NI = A, NS = A, NN = G/A, NK = G). Differing from the Cas9 endonuclease of the CRISPR/Cas9 system, the FokI endonuclease acts as a dimer. The catalytic FokI domain is fused C-terminally to the right and left TALEN arms. Two binding sites of 16–20 bp are involved, separated by a spacer of around 15 bp lengths, which significantly enhances the sequence specificity. The right and left TALEN arms bind in a tail-to-tail orientation enabling the dimerization of the FokI domains such that the dimer is able to cut the DNA in the spacer region and generates sticky ends. In the CRISPR/Cas9 system, a shorter target DNA sequence together with a mismatch tolerance at the 5′ end of the sgRNA increases the risk of generating off-targets [27]. Restrictions for the target site design differ as the TALEN arms require only a 5′ thymine [30] whereas CRISPR/Cas9 target sites need the presence of a 3′ NGG (or NAG) PAM sequence [21]. CRISPR/Cas9 offers the advantage that cloning can be rapidly conducted by a PCR-approach. Meanwhile, generation of the TALEN arms by ligation of repetitive modules has been simplified by the availability of efficient novel assembly techniques [31–33].

Over the last few years, the accumulated experience with different genome-editing techniques revealed that each technique has its specific, inherent advantages as well as shortcomings [24–26, 34]. The availability of a comprehensive toolbox for genome editing of the new basal land plant model system Marchantia would therefore be ideal and facilitate the selection of the best-suited approach for a specific genome-editing application. In this study, we compared the efficiencies of custom and assembled TALENs, which disrupt the Mp*NOP1* function. We demonstrate that Marchantia knockout mutants can be generated with an efficiency of over 20% and anticipate that this technique will further advance the use of this novel model organism to investigate the evolution of land plant gene functions.

## Methods

### Construction of custom TALEN vectors

**Fig. 1:**
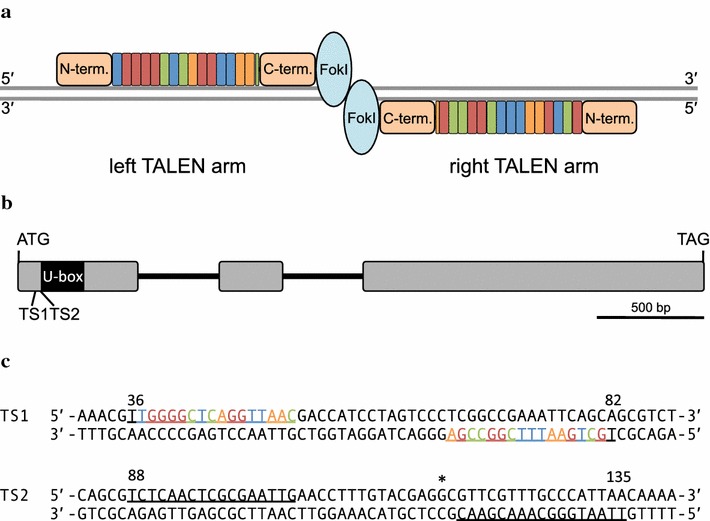
TALEN design for targeting Mp*NOP1.* **a** Schematic depiction of left and right TALEN arms binding to a target site. Four basic 33–35 aa long variable repeats, depicted as *blue* (T), *red* (G), *green* (C), and *orange* (A) *boxes*, bind via their RVDs to the four distinct nucleotides. The *color* code corresponds to the binding sites of the TS1. N-termini of the left and right TALEN arms contain a NLS. For generation of the TALEN, the FokI endonuclease is fused to the C-termini of the TALE proteins. FokI dimers introduce DSBs in the spacer region. **b** Mp*NOP1* locus with TS1 and TS2, localized 5′ of the crucial U-box. *Boxes*, exons; *lines*, introns. **c** Mp*NOP1* target sites TS1 and TS2 used for the two TALEN approaches. Nucleotides bound by TALEN proteins including the required 5′ T are *underlined*. 5′ and 3′ nt positions of the TS1 and TS2 sites are indicated above the sequences. *Asterisk* indicates start of the U-box

**Fig. 2:**
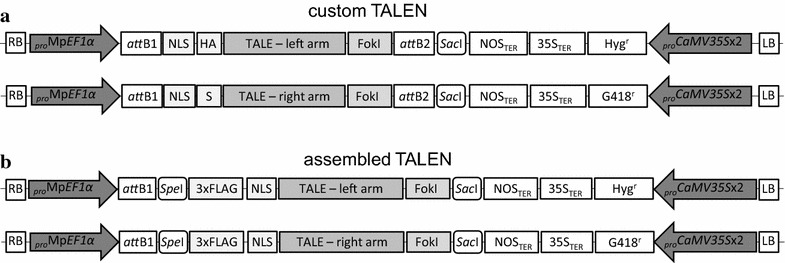
Structure of the T-DNA TALEN constructs. The left and right arm constructs of the custom TALENs (**a**) and assembled TALENs (**b**) were subcloned into pMpGWB103 and pMpGWB403 vectors, respectively. RB, LB, T-DNA left and right border; _*pro*_Mp*EF1α*, Mp*EF1α* promoter; *att*B, residual attachment site from Gateway cloning; NLS, nuclear localization signal; HA, hemagglutinin tag; S, S-peptide epitope; TALE, Transcription activator-like effectors; FokI, DNA cleavage domain of the FokI endonuclease; NOS, nopaline synthase; Ter, terminator; 35S, *CaMV35S* promoter; Hyg^r^, hygromycin resistance gene; G418^r^, G418 resistance gene; *Spe*I, *Sac*I, restriction sites for subcloning; 3×FLAG, threefold FLAG-tag

For the custom TALEN approach, TALEN coding sequences were purchased from Cellectis Bioresearch (SAS Paris, France, Fig. 1a). The Mp*NOP1* (AB830886) target sites (TS) TS1 and TS2 (Fig. 1b, c) were selected in collaboration with Cellectis Bioresearch considering the requirement of a thymine preceding the 5′ end of the target sites. The custom TALEN arms belong to the second-generation design, for which the N-terminus has been shortened and a nuclear localization signal (NLS) has been added. Left arms contain the HA and right arms the S-peptide epitope. The TALEN coding sequences were delivered and cloned into a vector based on the pTAL.pLESS backbone, which lacks a suitable promoter. We used the Gateway^®^ Cloning Technology to insert the TALEN arms in the binary expression vectors pMpGWB103 and pMpGWB403 allowing a double selection for hygromycin (Hyr^r^) and geneticin (G418^r^) (Fig. 2a; [35]). These vectors comprise the efficient Mp*EF1α* promoter and have been successfully used in generating transgenic *M. polymorpha* plants [35, 36].

For subcloning, each TALEN arm was amplified with primers containing *att*B sites (5′-GGGGACAAGTTTGTACAAAAAAGCAGGCTTCATGGGCGATCCTAAAAAGAAACG-3′, 5′-GGGGACCACTTTGTACAAGAAAGCTGGGTCTTATCAGTCGGCCGCGAAGTTGATC-3′) and cloned into the entry vector pDONR207 using the Gateway^®^ Technology (Invitrogen, USA) following the manufacturer’s instructions. The constructs were verified by sequencing and the TALEN arms then cloned into the binary expression vectors via the Gateway^®^ LR recombination reaction.

### Construction of assembled FastTALE™ vectors

For assembly of the TALEN arms, the FastTALE™ TALEN assembly kit from Sidansai Biotechnology was employed according to the manufacturer’s instructions. To compare the custom-designed and assembled TALEN approaches, the same TS1 and TS2 binding sequences were used (Fig. 1). The TALEN assembly kit provides all required materials and reagents, except for the PCR sequencing primers and restriction enzymes for control digestions. The available plant destination vectors contain the *CaMV35S* promoter, which requires a subcloning step into the suitable Marchantia binary vectors pMpGWB103 and pMpGWB403 possessing the Mp*EF1α* promoter. The kit includes 172 modules of ready-to-use DNA fragments that code for different TALE repeats and the vectors pL23–pL26 for the left arm cloning, which comprise sequences coding for the last 3′ half-repeat binding A, T, C or G, respectively. Likewise, vectors pR15–pR18 contain the last 3′ half-repeat binding A, T, C or G for the right arm. The procedure is summarized exemplarily for the left TALEN arm binding to the Mp*NOP1* TS1 (5′-TTGGGGCTCAGGTTAAC-3′, Fig. 1c). The first 5′ T as well as the last 3′ base of the target site are present in the destination vector and are thus not required as modules for the assembly procedure. The modules T1, G2, G3, GG4, CT5, CA6, GG7, TT8 and AA9 for the remaining binding sequences were selected according to the suppliers’ instructions. As the last required 3′ base of the left arm binding-site is a cytosine, vector pL25 was included in the ligation reaction. Likewise, target sequences for the other arms were divided into modules and inserted into vectors pR15 for the right arm of TS1 and into pL26 and pR17 for TS2, respectively. All selected modules were simultaneously assembled and inserted in the provided Ptalen vectors which harbor the *CaMV35S* promoter, requiring about 20 min bench time and in total about 5 h. Reactions were transformed into the supplied D18 *E. coli* cells and positive colonies were identified by PCR. Plasmids from positive clones were analyzed by using the unique restriction sites *Sac*I/*Spe*I together with *Sph*I to generate recognizable, diagnostic fragments and further verified by sequencing (forward primer: 5′-CTCCCCTTCAGCTGGACAC-3′, reverse primer: 5′-AGCTGGGCCACGATTGAC-3′). To subclone the TALEN arms by conventional cloning, pMpGWB103 and pMpGWB403 vectors were modified such that an additional *Spe*I restriction site was introduced 5′ of a *Sac*I restriction site. The assembled TALEN constructs were digested with *Spe*I/*Sac*I/*Sph*I and ligated into the modified binary vectors (Fig. 2b). The resulting expression vectors were verified by restriction digests and by sequencing (forward primer: 5′-GGGGACAAGTTTGTACAAAAAAGCAGGCT-3′, reverse primer: 5′-CTCATAAATAACGTCATGCATTAC-3′).

### Marchantia growth and transformation

Experiments were performed using the *M. polymorpha* ecotype BoGa, which was obtained from the Botanical Garden of Osnabrück, Germany. *Agrobacterium tumefaciens* mediated transformation of sporelings with two constructs was carried out as previously described by Ishizaki et al. [10] with minor modifications. Differing from Ishizaki et al. [10], the separation of sporelings from *A. tumefaciens* after three days of co-cultivation was conducted by a 15 min sedimentation followed by decantation of the added washing solution (½ Gamborg B5 medium with vitamins, Duchefa). Sedimentation and decantation were repeated four times and the sporelings were then plated on agar plates with ½ Gamborg B5 medium with vitamins supplemented with 10 µg/ml hygromycin (Sigma-Aldrich), 5 µg/ml G418 (Sigma-Aldrich) and 100 µg/ml cefotaxime (Duchefa). Gemmae were investigated from the G1 up to the G4 generation. Plants were grown under sterile conditions on solid medium (1% agar medium and ½ Gamborg B5 medium with vitamins, Duchefa) in 10 × 10 × 2 cm petri dishes (Sarstedt, Nümbrecht, Germany) under a 16 h/8 h day/night regimen at 22 °C in climate cabinets (Sanyo MLR351).

### Analyses of transgenic lines

Mp*nop1* knockout mutants were identified by phenotypic screening of the T1 plants. T1 plants represent the first generation of thalli, which develop from the transformed sporelings. Transgenic lines were investigated under the stereomicroscope (Leica M165 FC) to confirm the lack of air chamber formation and photographed with an integrated Leica DFC490 camera. In total, 24 plants exhibiting the mutant phenotype after 5 weeks were selected for sequencing using the primer 5′-GACAGGGAAGACTTTGGAGAG-3′. Genomic DNA (gDNA) was extracted from each line according to Edwards et al. [37]. The gDNA served as a template to amplify a 440 bp fragment of the Mp*NOP1* locus containing the TALEN target sites (5′-ATGGTCGATGTGATTGGTAATATTTTTG-3′, 5′-GATCTACACGCCCTTTGCTTGAG-3′). The stability of the genomic modifications was investigated in six selected Mp*nop1* ^*ge*^ lines by continuous phenotype analysis and sequencing of the target sites from two lines up to the forth gemmae generation.

## Results

### Targeted TALEN mutagenesis strategy

To establish an efficient TALEN technique in *M. polymorpha*, two different TALEN approaches were conducted. To reduce the labor-intensive and time-consuming multiple cloning steps of the different TALEN RVDs, we compared purchased custom TALENs from Cellectis with self-assembled TALENs. Several TALEN assembly kits are commercially available and two have been successfully applied for plant gene targeting. The Golden Gate method (Addgene) has been used for assembling multiple DNA fragments in an ordered fashion via custom repeat arrays in about three days and has enabled the targeting of genomic sites in rice [31]. We applied the FastTALE™ TALEN assembly kit (Sidansai Biotechnology) for Marchantia, which has been employed to generate an efficient targeted mutagenesis in soybean [32]. This kit offers the advantage that TALEN repeat arrays can be constructed by a one-step assembly and ligation procedure in only half a day.

**Fig. 3:**
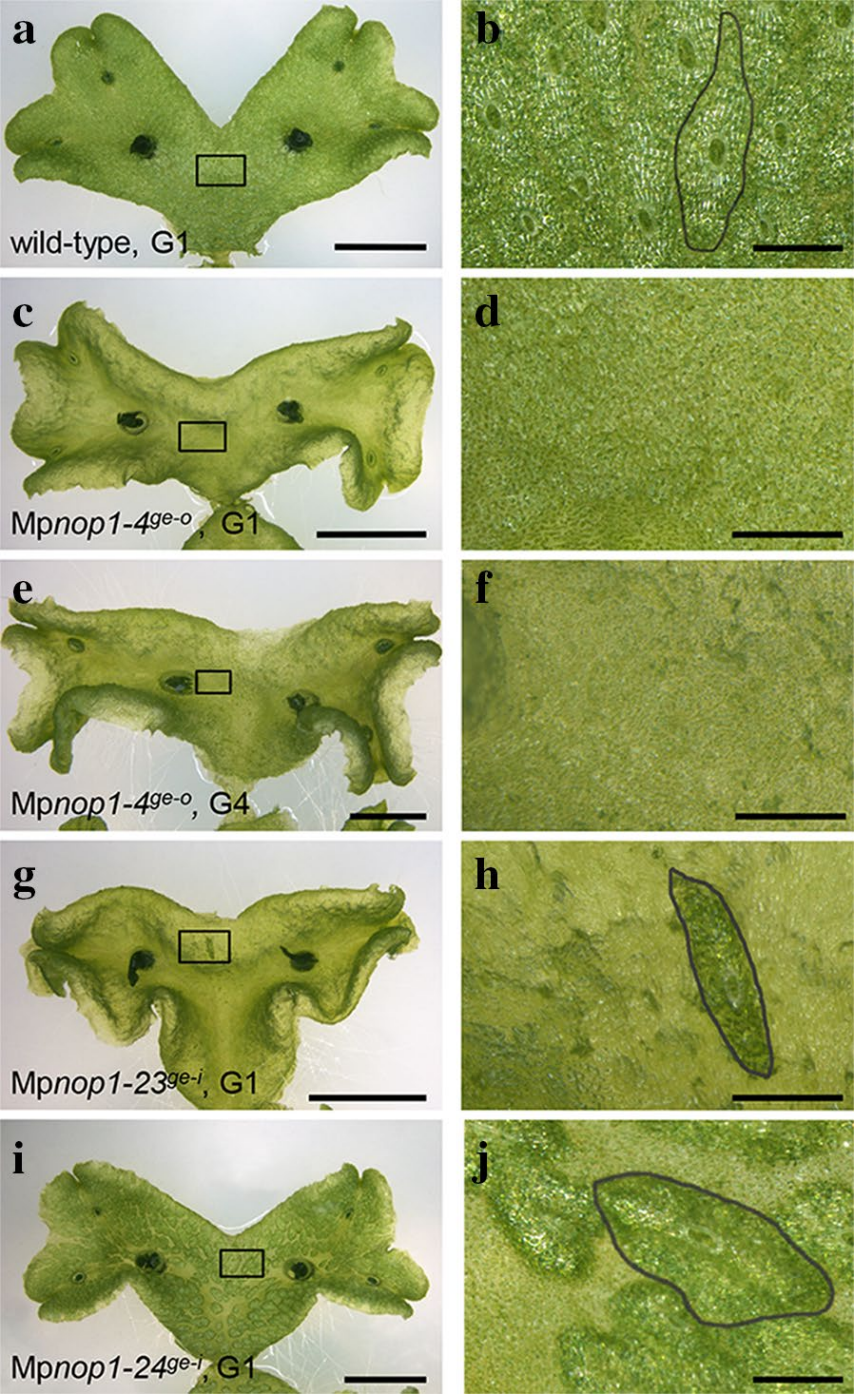
Phenotypes of TALEN-induced Mp*nop1* mutations. *Left panel* shows 4 week-old thalli of G1 plants (**a**, **c**, **g**, **i**) and one G4 plant (**e**) propagated vegetatively through clonal gemmae. *Boxes* delineate the area chosen for close-up images depicted in **b**, **d**, **f**, **h**, ***j***. The typical Mp*nop1* kockout phenotype lacking air chamber formation is shown exemplarily for one G1 plant from the Mp*nop1*-*4* ^*ge*-*o*^ line (**c**, **d**). The knockout phenotype was maintained without alterations throughout further G2 and G3 generations till G4 (**e**, **f**). Two lines with in-frame genome modifications show deviations from the typical Mp*nop1* mutant phenotype by producing only exceptionally (**g**, **h**, Mp*nop1*-*23* ^*ge*-*i*^) or less frequently irregularly shaped air chambers (**i**, **j**, Mp*nop1*-*24* ^*ge*-*i*^), indicated by encirclement of one air chamber, respectively. *Bars* **a**, **c**, **e**, **g**, **i** = 5 mm; **b**, **d**, **f**, **h**, **j** = 1 mm

To facilitate the detection of knockout mutants, we chose to target the Mp*NOP1* gene, as Mp*nop1* mutants exhibit an easily identifiable phenotype [16]. Wild-type Marchantia plants develop regularly distributed air chambers on their dorsal thallus surface and each air chamber forms a central air pore, a stomata-like structure (Fig. 3a, b). In Mp*nop1* mutants, general thallus growth is not affected but air chamber formation is impaired and air pores do not develop. Instead, a single continuous epidermal cell layer comprising chloroplasts is formed. As Mp*nop1* plants lack the numerous chlorophyll-rich filamentous cells formed within the air chambers, their thalli have a slightly more transparent appearance (Fig. 3c–f, [16]). Right and left TALEN arms were designed for two distinct Mp*NOP1* target sites in order to compare gene-editing efficiencies for different targeted nucleotide sequences. Furthermore, in case of off-target activities, these should differ for the two target sites and generate different phenotypes. Mp*NOP1* encodes a plant U-box E3 ubiquitin ligase that exhibits its ligase activity in a U-box dependent manner [16]. NHEJ events should preferably occur before the U-box to avoid the generation of truncated MpNOP1 proteins that possess the crucial N-terminal U-box (Fig. 1b). Therefore, TS1 and TS2 were selected such that they start 36 and 88 bp downstream of the Mp*NOP1* start codon, respectively. For each target site, left and right TALEN arms bind to a 17 bp long target sequence and the respective spacer sequences comprise 15 bp for TS1 and 16 bp for TS2 (Fig. 1c).

### Generation of custom and assembled TALEN constructs

Both custom and assembled TALEN arms required subcloning into suitable binary vectors. The gateway compatible destination vectors pMpGWB103 and pMpGWB403 were used as final binary vectors for Marchantia transformation. Both vectors contain the Mp*EF1α* promoter, which is strongly expressed in Marchantia tissues including the meristematic regions and has successfully been used for the generation of transgenic *M. polymorpha* plants [35, 36]. Double selection with hygromycin and G418 enabled the selection of transgenic T1 plants carrying right and left TALEN arm integrations. The custom TALENs from Cellectis (Fig. 2a) belong to the improved second-generation, which has been shown to enhance the gene targeting efficiency in Arabidopsis [38]. To introduce the custom TALEN pairs into the binary Marchantia vectors, the right and left TALEN arms were first amplified from the supplied vectors using primers containing *att*B sites, subcloned into a Gateway-compatible entry vector and transferred into the pMpGWB103/403 vectors (Fig. 2a). The FastTALE™ TALEN arms were assembled and ligated into the provided expression vectors by a one-step-reaction within half a day. TALEN constructs were digested with *Spe*I/*Sac*I/*Sph*I and subcloned into modified pMpGWB103/403 destination vectors comprising an introduced *Sac*I site to express the TALEN arms under control of the Mp*EF1α* promoter (Fig. 2b).

### Analysis of transgenic T1 populations

After Agrobacterium-mediated sporeling transformation, plants were grown for 5 weeks under double selection conditions. Altogether, 392 surviving T1 plants were analyzed to identify transgenic plants revealing the impaired air chamber formation phenotype characteristic for the Mp*nop1* mutant (Table 1; [16]). 44% (20/45) of the TS1 custom TALEN plants displayed the Mp*nop1* mutant phenotype, which is exemplarily shown for all confirmed mutant lines in Fig. 3c, d. For the TS2 site, 19% (38/199) of the T1 plants revealed an impaired air chamber formation. The custom TALEN pairs were transformed three times to analyze the variability between independent transformations using identical constructs. The efficiencies were 71, 38 and 20% for TS1 and 21, 22 and 9% for TS2, demonstrating that over threefold efficiency differences can occur between independently conducted transformation experiments. One transformation per target site was conducted with self-assembled TALEN vectors. For TS1, 6% (4/64) and for TS2, 42% (35/84) of the T1 plants exhibited the Mp*nop1* knockout phenotype. Considering that both approaches used the same TS1 and TS2 sites, the overall TALEN mutagenesis showed a high efficiency of over 20%, reaching values of 22% for TS1 and 26% for TS2 (Table 1).

**Table 1:** Modification efficiency of the two TALEN approaches

TALEN	Custom						Assembled		Custom and assembled	
Target site	TS1			TS2			TS1	TS2	TS1	TS2
Experiment	A	B	C	A	B	C				
T1 plants	14	21	10	39	117	43	64	84	109	283
Mp*nop1*-like	10	8	2	8	26	4	4	35	24	73
Efficiency (%)	71	38	20	21	22	9	6	42	22	26

Three independent transformations were conducted for each custom TALEN target site (experiment A–C) and one for each assembled TALEN target site. In total, 392 T1 plants were analyzed. Efficiencies were determined by identification of T1 plants exhibiting the Mp*nop1* knockout phenotype

**Fig. 4:**
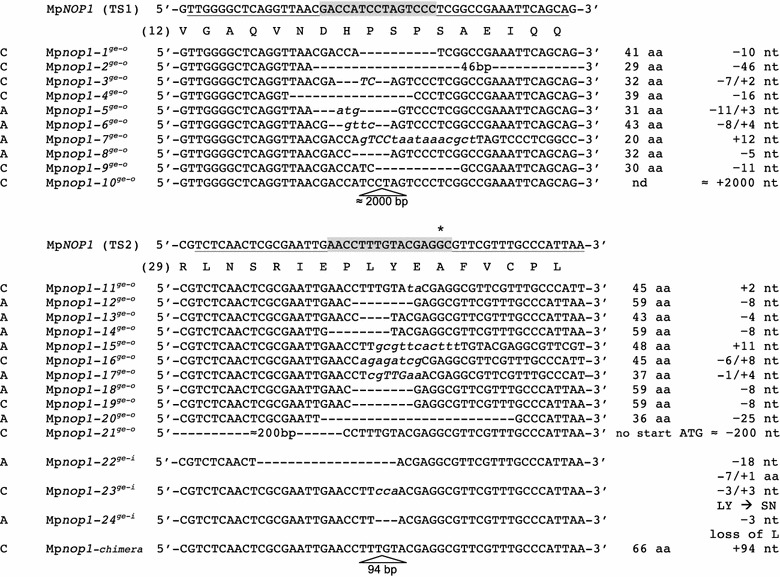
Nucleotide modifications in the Mp*NOP1* locus induced by TALENs. Alignment of analyzed Mp*NOP1* TS1 and TS2 sequences from T1 mutants. The respective wild-type nucleotide and amino acid Mp*NOP1* sequences are depicted at the *top* of each target site. *Numbers* in *brackets* indicate the position of the first indicated amino acid. TALEN binding sites are *underlined* and spacer sequences are highlighted in *grey*. Origin of the TALEN pairs is indicated using C for custom TALENs (Cellectis) and A for the assembled TALENs (Sidansai) on the *left side*. Deletions in the genomic locus are indicated by *dashes*, insertions by *italicized lowercase letters*. Amino acid changes in out-of-frame and in-frame mutants are indicated by the *suffixes* ^*ge*-*o*^ and ^*ge*-*i*^. The predicted translated protein length till occurrence of a novel, artificial stop codon in the ^*ge*-*o*^ mutants is shown on the *right side* along with the length of the deletions (−) and insertions (+). *Asterisk* indicates A40 as putative start of the U-box; *nd* not determined

For further analysis, 22 T1 plants of either approach (10 for TS1, 12 for TS2) exhibiting the Mp*nop1* mutant phenotype were selected and sequenced to confirm the genome-editing event of TS1 and TS2 and also to determine the genetic modifications that occurred by imprecise NHEJ repair of the cleaved genomic Mp*NOP1* locus. Corroborating the mutant phenotype, 21 of the investigated 22 lines were altered such that open reading frame shifts occurred, indicated by the suffix ^*ge-o*^. The generation of premature stop codons likely led to the formation of truncated, non-functional MpNOP1 proteins (Fig. 4). 13 of the 22 T1 lines harbored deletions at the target sites varying between 4 nt up to 2000 nt. Most frequently, in four lines, 8 nt deletions were observed. Four insertion lines were obtained, where the most extreme case is represented by Mp*nop1*-*10* ^*ge*-*o*^ which carries an integration of over 2000 nt. Five lines with a combination of deletions and insertions were detected. Noteworthy, Mp*nop1*-*22* ^*ge*-*i*^ carried an in-frame deletion (^*ge*-*i*^) of 18 nt, suggesting that the absence of six amino acids directly upstream of the U-box severely affects the MpNOP1 protein activity in air chamber formation. These findings are consistent with previous reports, where TALEN approaches generated a variety of sequence modifications in plants [38–40].

Interestingly, two of the 392 T1 lines showed novel phenotypes. In contrast to a full Mp*nop1* knockout lacking all air chambers, Mp*nop1*-*23* ^*ge*-*i*^ formed only exceptionally single air chambers (Fig. 3g, h) and Mp*nop1*-*24* ^*ge*-*i*^ developed a reduced number of air chambers with a more irregular shape (Fig. 3i, j). Sequencing analysis by testing different thallus areas from each plant revealed that these lines are not chimeras but that gene targeting generated in-frame changes at TS2. L37 and Y38 were replaced by S37 and N38 in Mp*nop1*-*23* ^*ge*-*i*^ and L37 was deleted in Mp*nop1*-*24* ^*ge*-*i*^ (Fig. 4), emphasizing the importance of the region upstream of the U-box for normal air chamber formation.

**Fig. 5:**
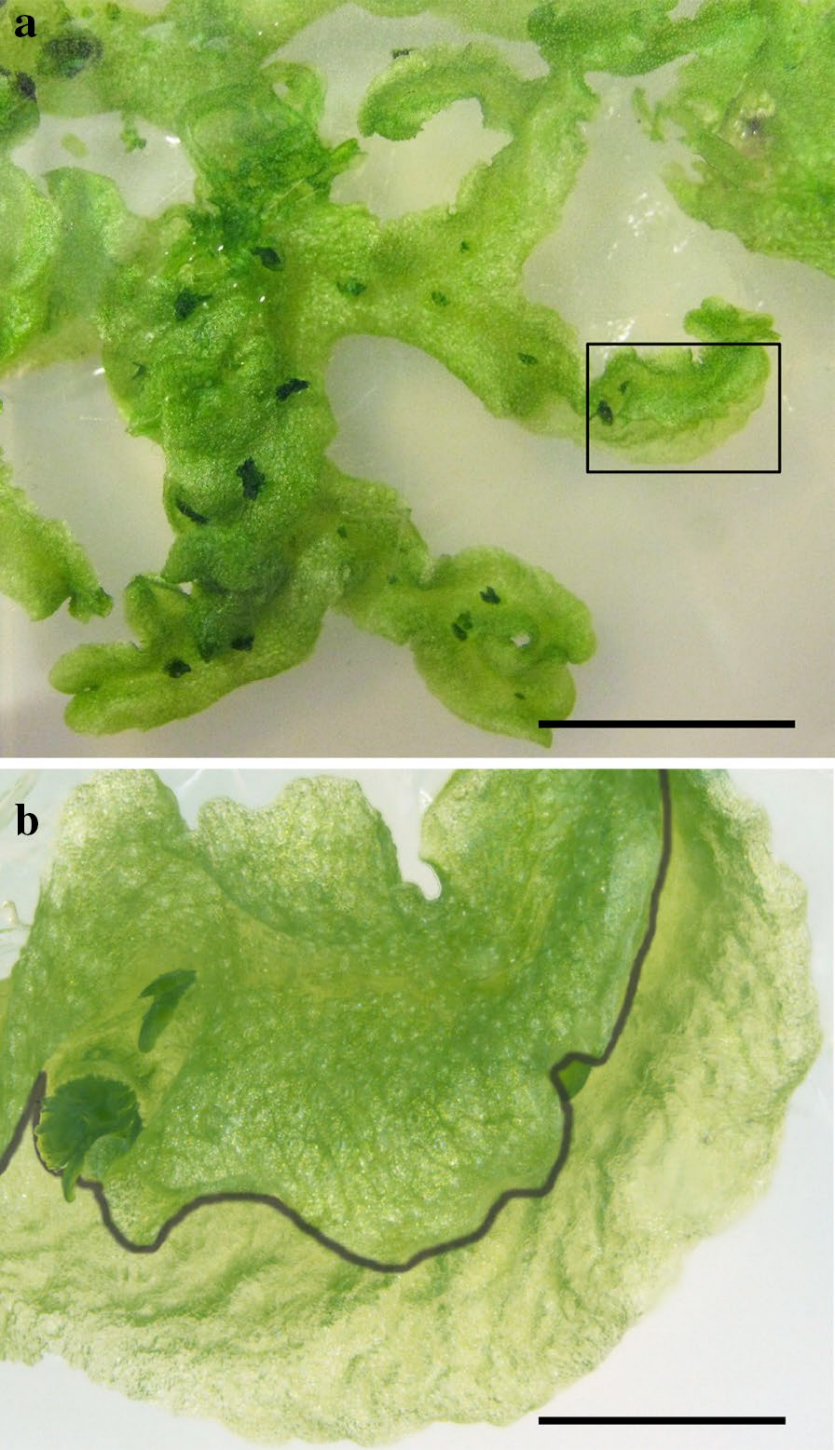
Chimeric Mp*nop1* mutant. One out of 392 7 week-old T1 plants showed a chimeric phenotype. **a** Overview of the chimeric T1 plant named Mp*nop1*-*chimera*. The *box* indicates the chimeric thallus lobe. **b** Detailed view of the chimeric thallus lobe, the *line* denotes the border between wild-type and Mp*nop1* mutant tissue. *Bars* **a** = 1 cm; **b** = 2 mm

To detect eventual phenotypic changes occurring later in the T1 lines, we continued to observe the development of all mutant and wild-type-like transgenic T1 lines. One initially wild-type-like plant showed after cultivation for 7 weeks a sector of Mp*nop1* mutant-like tissue on one site of one thallus arm (Fig. 5a, b). Distinct wild-type-like and Mpn*op1*-mutant-like tissues were harvested from this plant and sequenced. This revealed a 94 nt insertion in TS2 in the Mpn*op1*-like tissue, whereas the wild-type-like sector possessed the wild-type Mp*NOP1* sequence. The different tissue parts of the chimeric lobe from this late-formed Mp*nop1*-*chimera* line were transferred to plates with new medium. None of the newly formed thalli exhibited a mosaic phenotype and each tissue developed according to its origin either in a wild-type-like or Mp*nop*-*like* plant. As the T1 lines were cultivated continuously on the original selection media, the mosaic phenotype might have resulted from a late transformation event caused by residual *A. tumefaciens*, which can be impeded by transferring T1 plants to new plates supplemented with antibiotics to restrict *A. tumefaciens* growth.

### Stability of TALEN-induced mutations throughout gemmae generations

Marchantia offers the advantage to propagate transgenic T1 plants asexually via clonal propagation of their gemmae, the G1 plants. These isogenic gemmae are produced from single initial cells in the T1 gemmae cups on the dorsal surface of the thallus [1]. To investigate the stability of the mutations throughout successive G1, G2, G3 and G4 generations, five Mp*nop* ^*ge*-*o*^ lines were further analyzed. For each target site we selected from the custom and assembled TALEN approach at least one verified Mp*nop1* knockout plant, namely Mp*nop1*-*4* ^*ge*-*o*^, -*5* ^*ge*-*o*^, -*11* ^*ge*-*o*^, -*12* ^*ge*-*o*^, -*13* ^*ge*-*o*^. Additionally, one line harboring an in-frame mutation, Mp*nop1*-*22* ^*ge*-*i*^, was also further analyzed. All six lines retained the knockout mutant phenotype and exhibited no reversions or additional phenotypes in the four analyzed gemmae generations (Fig. 3e, f). Consistency of the unaltered target sites was further confirmed by sequence analysis of two G4 lines.

## Discussion

### Efficiency of the two Marchantia TALEN approaches

Genome editing using TALENs has been employed in several plant species and efficiencies of over 20% and occasionally up to 70% have been reported [31, 32, 39, 41, 42]. In this study, analysis of two TALEN approaches targeting the Mp*NOP1* gene revealed in 392 T1 plants an overall efficiency of 22% for TS1 and 26% for TS2. Our results demonstrate that the application of the Mp*EF1α* promoter driving the expression of custom TALENs from Cellectis as well as assembled TALENs using the FastTALE™ TALEN assembly kit generated highly efficient promoter-TALEN combinations. The establishment of the first targeted Marchantia knockout technique by HR succeeded in the generation of approximately 3% mutants in T1 populations [15]. For the recently established Marchantia CRISPR/Cas9 genome-editing technology, the sgRNA was expressed under the control of the endogenous Marchantia U6 polymerase III promoter to target Mp*ARF1,* coding for the *AUXIN RESPONSE FACTOR1*. This enabled a pre-selection step, as plants lacking Mp*ARF*1 activity fail to respond to synthetic auxin and thus do not form rhizoids. Analysis of a phenotypically pre-screened T1 population revealed that 11% of knockout mutants were obtained [29]. The high TALEN efficiencies obtained in this study enable the identification of transgenic plants that do not display visible phenotypes by sequencing 50–100 T1 plants. Ideally, these numbers can be further decreased given the possibility to incorporate pre-selection steps.

TALEN construction involves re-engineering a new protein for each target. The FastTALE™ kit enables the easy and rapid assembly and ligation of the TALEN arms within 5 h. In comparison, generation of plasmids using the GoldenGate TALEN repeat modules is conducted in several steps and requires approximately three days (http://www.addgene.org/TALeffector/goldengateV2/k, [43]). Custom and self-assembled TALEN techniques require subcloning of the TALEN arms into suitable Marchantia vectors, which is also necessary for the CRISPR/Cas9 approach [29], making the practicability of these two genome-editing techniques comparable with respect to time requirements. Recent comparisons between TALENs and CRISPR/Cas9 showed that depending on the application, both techniques have their specific benefits [34, 44, 45]. CRISPR/Cas9 Arabidopsis lines can be rapidly established but were often found to be mosaic in the T1 generation, emphasizing the necessity to identify appropriate promoters driving Cas9 expression in the required tissues [46–48]. Furthermore, pol III promoters such as the U6 promoter are commonly used to express the sgRNAs, which require a precise transcription start and termination for successful recognition by the Cas9 protein. Deep sequencing revealed that the identification of a suitable U6 promoter is critical in order to avoid an inaccuracy of 5′ sgRNA ends, as this might lead to formation of transcripts with variable lengths ultimately reducing the CRISPR/Cas9 editing efficiencies [49]. The TALEN approach does not rely on a correct sgRNA folding for endonuclease recognition but depends solely on the correct expression of the TALEN arm proteins. The Mp*EF1α* promoter has been employed for the generation of several successfully applied binary Marchantia vectors [35, 36] and was therefore applied to establish the TALEN technique in Marchantia.

The TALEN and CRISPR/Cas9 approaches both enable precise gene targeting, but off-target formation is a possible side effect. CRISPR/Cas9 achieves specificity through the sgRNA, which can tolerate up to five mismatches within an up to 20 nt long target site [27]. The TALEN strategy requires protein binding to two distinct sites, each approximately 16 bp in length, which are separated by an about 15 bp long spacer. Compared to the short CRISPR/Cas9 target site, such a sequence is less likely to occur elsewhere in the genome, which is supported by recent studies detecting extremely low TALEN off-target rates [39, 50]. Therefore, CRISPR/Cas9 is considered to be more prone to generate off-targets than TALENs [51]. In addition, by screening the barley genome, Gurushidze et al. [39] did not detect potential TALEN off-targets. Next generation sequencing of an Arabidopsis T2 plant generated by a custom TALEN approach revealed only three additional deletions, which were unrelated to the TALEN binding site and seemed to have occurred spontaneously [38]. TALEN off-target activities are, therefore considered to be extremely low and might not be distinguishable from the occurrence of unrelated, random mutations in the genome. We conducted two TALEN approaches for two distinct Mp*NOP1* target sites. In 392 analyzed T1 plants, no phenotypes besides Mp*nop1*-related ones were detectable. To exclude that TALEN off-target effects are hampering knockout mutant identification it is advisable to conduct the TALEN approach for at least two target sites.

Besides the different CRISPR/Cas9 and TALEN target site length requirements and coinciding specificity differences, the CRISPR sgRNA target site selection is constrained by the requirement of a 3′ NGG (PAM) site following the binding sequence, whereas TALEN pair design provides a higher target site flexibility as it requires only a 5′ thymine for efficient TALEN binding [20–22]. The shorter 20 nt long CRISPR binding sites are better suited for multiplex approaches to knock out several target sites of genes which share a high degree of sequence homology [52, 53]. This is particularly interesting for redundantly acting genes belonging to larger gene families. However, as the number of genes per family is exceptionally low in Marchantia compared to other land plants [9], the demand for such an application might be higher for investigations in angiosperm species.

### TALENs generate out-of-frame and in-frame mutations

Small indels ranging from 3 to 20 bp occurred most frequently in the analyzed G1 lines, similar to the reports from other TALEN approaches [31, 38, 39, 41, 42]. Sequencing revealed that 21 out of 22 lines are out-of-frame mutants. The occurrence of premature stop codons might cause the formation of truncated, non-functional MpNOP1 proteins not comprising the crucial U-box of the E3 ubiquitin-protein ligase. Also, aberrant mRNAs might be eliminated via the non-sense mediated mRNA decay mechanism which prevents the synthesis of truncated proteins [54]. Three lines carrying in-frame mutations localized before the predicted start of the MpNOP1 U-box (A40) showed interesting phenotypes. Although these lines likely still synthesize normal MpNOP1 protein levels, the mutations seem to interfere with the formation of a functional U-box. Mp*nop1*-*22* ^*ge*-*i*^ lacks six amino acids and resembles full Mp*nop1* knockout plants. The change of L37 and Y38 into S37 and N38 in Mp*nop1*-*23* ^*ge*-*i*^ leads to a knockout-like phenotype with an extremely rare air chamber formation. The loss of L37 in Mp*nop1*-*24* ^*ge*-*i*^ plants generates a less severe phenotype with reduced air chamber formation. The data emphasize the importance of the hydrophobic L37 for normal MpNOP1 activity, which might negatively affect folding of the downstream U-box. As CRISPR/Cas9 mutants predominately result from shorter deletions and insertions, larger deletions and in-frame mutations seem more likely to occur with the TALEN approach [46, 47]. This might be advantageous for the analysis of Marchantia genes, where full knockout mutants are lethal. Therefore, the TALEN approach is also suitable for generating gene modifications with reasonable frequencies such that an essential gene activity is not fully disrupted but allows insight into the function of crucial amino acids and/or motives.

## Conclusion

The TALEN technique involves two DNA binding regions, which reduces off-target effects and a relatively unrestricted design enables the targeting of almost any sequence. Different strategies, such as custom TALENs supplied by a company or self-assembled TALENs generated by kits can be employed to generate the two TALEN arms. Both strategies require the same time for further subcloning into destination vectors harboring suitable promoters. Here, we show that TALEN constructs can be produced within only half a day using the FastTALE™ assembly kit, which provides a convenient and rapid alternative to commercial TALENs. In Marchantia, homologous recombination yielded 3% knockout mutants [15] and the CRISPR method generated 11% mutants in a T1 population that has been pre-screened by a positive selection step [16]. Given the obtained targeting efficiencies of over 20% for the Mp*NOP1* gene, the TALEN approach is a highly efficient tool for Marchantia knockout mutant generation. Optimizing the efficiencies of different genome-editing techniques further will provide a toolbox for the selection of the best-suited approach for individual advanced applications. Besides knockout mutant generation, this will enable future genomic DNA replacements to be conducted and facilitates the introduction of protein tags for tracing endogenous gene expression to elucidate gene functions in the novel basal land plant model system *Marchantia polymorpha*.
